# First-Principles Study of the Structural, Mechanical, Electronic, and Thermodynamic Properties of AlCu_2_M (M = Ti, Cr, Zr, Sc, Hf, Mn, Pa, Lu, Pm) Ternary Intermetallic Compounds

**DOI:** 10.3390/ma17143441

**Published:** 2024-07-11

**Authors:** Yu Guo, Bo Jiang, Xun Zhang, Shikang Li

**Affiliations:** 1 Huzhou Key Laboratory of Green Energy Materials and Battery Cascade Utilization, Huzhou College, Huzhou 313000, China; 2 School of Intelligent Manufacturing, Huzhou College, Huzhou 313000, China; 3 School of Materials Science and Engineering, Harbin University of Science and Technology, Harbin 150004, China; 4 School of Architectural Engineering, Harbin University of Science and Technology, Harbin 150004, China

**Keywords:** aluminides, mechanical properties, thermodynamic properties, first-principles theory

## Abstract

Based on the first principles, the structural stability, mechanical characteristics, electronic structure, and thermodynamic properties of AlCu_2_M (M = Ti, Cr, Zr, Sc, Hf, Mn, Pa, Lu, Pm) are investigated. The calculated results indicate that the AlCu_2_Pa crystal structure is more stable and that AlCu_2_Pa should be easier to form. All of the AlCu_2_M compounds have structural stability in the ground state. Elastic constants are used to characterize the mechanical stability and elastic modulus, while the B/G values and Poisson ratio demonstrate the brittleness and ductility of AlCu_2_M compounds. It is demonstrated that all computed AlCu_2_M compounds are ductile and mechanically stable, with AlCu_2_Hf having the highest bulk modulus and AlCu_2_Mn having the highest Young’s modulus. AlCu_2_Mn has the highest intrinsic hardness among AlCu_2_M compounds, according to calculations of their intrinsic hardness. The electronic densities of states are discussed in detail; it was discovered that all AlCu_2_M compounds form Al-Cu and Al-M covalent bonds. Additionally, we observe that the Debye temperature and minimum thermal conductivity of AlCu_2_Mn and AlCu_2_Sc are both larger than those of others, indicating stronger chemical bonds and higher thermal conductivities, which is consistent with the elastic modulus results.

## 1. Introduction

High specific strength and superior thermal conduction have made high-performance Al-Cu-based alloys popular in the microelectronics, new-energy vehicles, and aerospace industries [1,2,3]. However, more research on Al-Cu alloys is needed as the need for lightweight materials with increased heat conductivity and mechanical strength grows. Al-Cu’s strength and heat conductivity can generally be impacted by the addition of alloying elements. As a result, micro-alloying can give Al-Cu alloys a useful means of achieving high conductivity and strong mechanical properties [4,5].

Owing to the exceptional performance of Al-Cu alloys, additional research has concentrated on enhancing their characteristics via theoretical calculations and experimental studies. Based on the excellent performance of Al-Cu alloys, more studies have focused on improving their properties through experimental investigations and theoretical calculations. Song et al. [6] have suggested that the co-addition of rare earth elements Sc and La can significantly increase the ultimate tensile strength, yield strength, and thermal conductivity, which are higher than in Al-4.8Cu alloy. The investigation results show that elements Hf and Si play a role in promoting ordered precipitates, and metastable Al-Cu intermetallic compounds θ″/θ′ have been subjected to quantitative studies [7]. The addition of Mn with Zr/Ti can play a significant role in stabilizing the metastable θ′ precipitates responsible for the alloy’s hardness in high-temperature applications, and density functional theory calculations have shown that the co-precipitate is a key factor that governs microstructural stability beyond 300 °C [8]. Khisamov et al. [9] have used the density functional theory approach to calculate the work function (WF) values of AlCu, Al_2_Cu, and Al_4_Cu_9_ intermetallic phases and compared them to the experimentally measured WF values of the Al-Cu metal matrix composite. Furthermore, ternary intermetallic compounds can be created by adding transition and rare-earth elements, and some investigations based on density-functional theory have been conducted on these compounds [10,11,12,13], which can offer a theoretical foundation for the practical application and quantitative design of Al-Cu alloys.

In this work, we have calculated the structural, mechanical, electronic, and thermodynamic properties of AlCu_2_M (M = Ti, Cr, Zr, Sc, Hf, Mn, Pa, Lu, and Pm) ternary intermetallic compounds using first principles based on the density functional theory. Mechanical properties such as elastic constants, elastic modulus, Pugh’s criteria, and Poisson’s ratio have been investigated for AlCu_2_M compounds. The density of the state is used to illustrate the electronic structures and inter-atomic bonding of AlCu_2_M compounds. Furthermore, the thermodynamic properties are revealed by estimating their Debye temperatures and minimum thermal conductivity.

## 2. Computational Methods

The structural, electronic, elastic, and thermodynamic characteristics of AlCu_2_M (M = Ti, Cr, Zr, Sc, Hf, Mn, Pa, Lu, and Pm) compounds were assessed using density functional theory (DFT) based on the framework of the Cambridge Sequential Total Energy Package (CASTEP) code [14,15], as implemented in the plane wave pseudo-potential method. The electronic exchange correlation energy of the structural optimization of all AlCu_2_M compounds was described within Perdew–Burke and Ernzerhof (PBE) using generalized gradient approximation (GGA) [16]. The valence electron configurations for the constituent elements of AlCu_2_M (M = Ti, Cr, Zr, Sc, Hf, Mn, Pa, Lu, and Pm) compounds correspond to Al (3s^2^ 3p^1^), Cu (3d^10^ 4s^1^), Ti (3s^2^ 3p^6^ 3d^2^ 4s^2^), Cr (3s^2^ 3p^6^ 3d^5^ 4s^1^), Zr (4s^2^ 4p^6^ 4d^2^ 5s^2^), Sc (3s^2^ 3p^6^ 3d^1^ 4s^2^), Hf (4f^14^ 5s^2^ 5p^6^ 5d^2^ 6s^2^), Mn (3s^2^ 3p^6^ 3d^5^ 4s^2^), Pa (5f^2^ 6s^2^ 6p^6^ 6d^1^ 7s^2^), Lu (4f^14^ 5s^2^ 5p^6^ 5d^1^ 6s^2^), and Pm (4f^5^ 5s^2^ 5p^6^ 6s^2^), respectively.

The structures were optimized by geometry optimization with suitable convergence parameters. The plane wave energy cut-off was employed in all PBE calculations via the Kohn–Sham function, which was set to 440 eV for AlCu_2_M (M = Ti, Cr, Zr, Sc, Hf, Mn, Pa, and Lu) and 480 eV for AlCu_2_Pm, respectively. The k-point meshes for the Brillouin zone (BZ) were set within the Monkhorst–Pack scheme [17] with 4 × 4 × 4 grids for AlCu_2_M compounds. The Broyden–Fletcher–Goldfarb–Shanno (BFGS) optimization algorithm was utilized to obtain a structure with the lowest energy [18]. The interactions of ionic core and valence electrons were described by ultrasoft pseudo-potentials in DFT. The following parameters were displayed in all relaxation calculations: the ionic Hellmann–Feynman force [19] acting on each atom was less than 0.01 eV/Å, the self-consistent convergence of total energy was reached at 5 × 10^−6^ eV, the maximum stress in the unit cell was less than 0.02 GPa, and the maximum ionic displacement was set at 5 × 10^−4^ Å. During geometry optimization, collinear spin polarization parameters determined the ferromagnetism of AlCu_2_Cr and AlCu_2_Mn. Furthermore, the quasi-harmonic Debye model was employed to study the thermodynamic properties of the AlCu_2_M compounds.

## 3. Results and Discussion

### 3.1. Structural Properties

**Figure 1:**
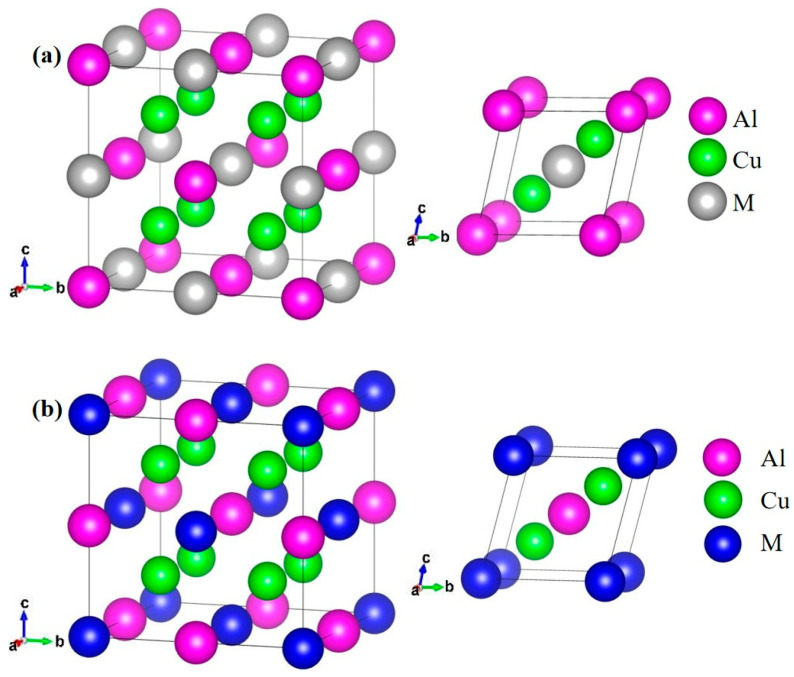
The crystal structure of the AlCu_2_M unit cell and primitive cell: the pink balls are Al atoms, green balls are Cu atoms, gray balls are M (M = Ti, Cr, Zr, Sc, Hf, Mn), (**a**) and blue balls are M (M = Pa, Lu, Pm) (**b**), respectively.

The space group of AlCu_2_M intermetallic compounds is Fm-3m (space group number: 225), and M (M = Ti, Cr, Zr, Sc, Hf, Mn, Pa, Lu, and Pm) is bonded in a body-centered cubic geometry to eight equivalent Cu atoms. Cu is bonded in a body-centered cubic geometry to four equivalent M and four equivalent Al atoms. Al is bonded in a body-centered cubic geometry to eight equivalent Cu atoms. The crystal structure of AlCu_2_M (M = Ti, Cr, Zr, Sc, Hf, Mn) is illustrated in Figure 1a, in which Al atoms occupy the 4a Wyckoff position, Cu atoms occupy the 8c Wyckoff position, and M atoms occupy the 4b Wyckoff position in the unit cell, respectively. The crystal structure of AlCu_2_M (M = Pa, Lu, Pm) is shown in Figure 1b, where M atoms occupy the 4a Wyckoff position, Cu atoms occupy the 8c Wyckoff position, and Al atoms occupy the 4b Wyckoff position in the unit cell, respectively.

The lattice constants (α), equilibrium volume (V_0_), and formation energy ΔH for AlCu_2_M compounds were calculated and are listed in Table 1. The calculated results of AlCu_2_M compounds with complete geometry optimization were compared with other experimental and theoretical results from Refs. [10,11,20,21,22,23,24,25]. We observed that the calculated values of α and V_0_ in this work are reasonably close to the experimental and theoretical values in the references.

The formation energy can represent the alloying abilities of AlCu_2_M compounds and can also indicate the crystal structure’s stability. When the value of formation energy is less than zero, the smaller the value of formation energy is, the more stable the crystal structure is [26]. The formation energy of AlCu_2_M compounds at zero temperature can be calculated by the following equation [27]:

(1) $$\Delta H=\frac{E_{tot}-N_{Al}E_{Solid}^{Al}-N_{Cu}E_{Solid}^{Cu}-N_{M}E_{Solid}^{M}}{N_{Al}+N_{Cu}+N_{M}}$$

where *E_tot_* is the total energy of AlCu_2_M compounds; $E_{Solid}^{Al}$, $E_{Solid}^{Cu}$ and $E_{Solid}^{M}$ are the energies per atom of Al, Cu, and M (M = Ti, Cr, Zr, Sc, Hf, Mn, Pa, Lu, Pm) in solid states; and *N_Al_*, *N_Cu_*, and *N_M_* denote the number of Al, Cu, and M atoms, respectively. These calculated results are also listed in Table 1, which demonstrates that all AlCu_2_M compounds have a negative Δ*H* and structural stability. It also indicates that elements M (M = Ti, Cr, Zr, Sc, Hf, Mn, Pa, Lu, and Pm), Al, and Cu have a strong alloying ability, and all AlCu_2_M compounds have stable crystal structures. More negative formation energy implies higher stability; thus, the stability of AlCu_2_M compounds can be sequenced as AlCu_2_Pa < AlCu_2_Cr < AlCu_2_Zr < AlCu_2_Mn < AlCu_2_Hf < AlCu_2_Ti < AlCu_2_Lu < AlCu_2_Sc < AlCu_2_Pm. This suggests that AlCu_2_Pa has far stronger chemical bonds than the other compounds, implying that AlCu_2_Pa will form more readily from the lowest formation energy.

### 3.2. Mechanical Properties

To investigate the mechanical properties of the AlCu_2_M compounds, the elastic constants *C_ij_* were calculated via a strain–stress function based on Hooke’s law [28]. The elastic constants directly reflect mechanical stability. The mechanical stability of AlCu_2_M compounds can be judged by independent elastic constants. For the cubic crystal structure, there are three independent elastic constants (*C*_11_, *C*_12_, and *C*_44_), and the mechanical stability requirements for cubic crystals are given as follows [29]:

(2) $$\left\{\begin{array}{l} C_{11}-\left|C_{12}\right|>0\\ C_{11}+2C_{12}>0\\ C_{11}>0\\ C_{44}>0 \end{array}\right\}$$

The calculated elastic constants of AlCu_2_M compounds in the ground state are listed in Table 2. The results are consistent with the other calculated report, with a difference of about 10% to 30%. It is shown that the calculated elastic constants *C*_12_ and *C*_44_ of AlCu_2_Hf calculated in this work are significantly larger than the theoretical values in Ref. [10] from the Vienna ab initio Simulation Package (VASP), whereas Ref. [10] determined the total energies by imposing appropriate strains up to ±2% at 0.5% intervals for each structure. Based on the mechanical stability criteria, the calculated elastic constants of all AlCu_2_M compounds (M = Ti, Cr, Zr, Sc, Hf, Mn, Pa, Lu, and Pm) meet the above criteria, showing mechanical stability. The mechanical stability of AlCu_2_M compounds (M = Ti, Cr, Zr, Sc, Hf, Mn, Pa, Lu, and Pm) is consistent with the corresponding formation energy calculations. Upon further analysis, the mechanical stability results agree well with the theoretical results reported in the literature [10,11,30].

According to the Voigt–Reuss–Hill (VRH) approximation method, the bulk modulus *B* and shear modulus *G* of the AlCu_2_M compounds can be estimated from the results of elastic constants [31,32,33]. Furthermore, the Young’s modulus *E* and Poisson’s ratio *ν* for each compound are calculated based on *B* and *G*, which can be obtained by the following formulas [34]:

(3) $$E=\frac{9B\times G}{3B+2G}$$

(4) $$\nu =\frac{3B-2G}{6B+2G}$$

Table 3 shows the calculated results of the elastic modulus, Poisson’s ratio, *B*/*G* value, and hardness for AlCu_2_M compounds. To verify the correctness of the calculations, the elastic moduli, Poisson ratios, and B/G of AlCu_2_M compounds are calculated and compared with the references [10,11,35]. The average error of the data is significant because our calculation results are compared with the other theoretical values with different calculation parameters and methods. The elastic modulus plays an important role in the mechanical properties of solids. The bulk modulus *B* is used to measure the resistance to volume deformation resulting from applied pressure [36]. From Table 3, it can be seen that the bulk modulus *B* of AlCu_2_Hf has the maximum value, and the *B* of AlCu_2_Pm decreases quickly. AlCu_2_Hf is stated to have the strongest resistance to deformation. In addition, it can be concluded that AlCu_2_Hf, AlCu_2_T, AlCu_2_Zr, AlCu_2_Sc, and AlCu_2_Mn are more incompressible than other AlCu_2_M compounds. The Young’s modulus *E* is used to represent the ability to resist tension and compression in the region of elastic deformation [37]. According to the calculated values in Table 3, the larger the Young’s modulus of AlCu_2_Mn, the more difficult it is to deform in the alloy; AlCu_2_Mn can be regarded as a strengthening phase [34].

Based on Pugh’s criterion, the ratio of *B*/*G* can be used to analyze the brittleness and ductility of AlCu_2_M compounds [38]. The *B*/*G* results for AlCu_2_M compounds are displayed in Table 3. When the *B*/*G* ratio is greater than 1.75, it indicates that the materials exhibit ductile behavior, and a material with *B*/*G* < 1.75 will be brittle. It is shown that all the calculated AlCu_2_M compounds in this work are ductile, with values greater than 1.75. Concurrently, AlCu_2_Mn has the lowest *B*/*G* value and the weakest ductility of any compound. Poisson’s ratios reflect crystals’ resistance to shear stress [39], and the calculated results are also listed in Table 3. All Poisson’s ratios of AlCu_2_M compounds are greater than 0.26. The greater the Poisson’s ratio, the softer the material, and it is demonstrated that AlCu_2_Pm is the softest of them all.

The intrinsic hardness is an important index with which to characterize the mechanical performance of AlCu_2_M compounds. Based on Tian’s theoretical hardness model (*H_VT_*), the hardness of AlCu_2_M compounds can be calculated via the following equation [40]:

(5) $$H_{VT}=0.92k^{-1.137}G^{0.708}$$

where *k* is the value of *B*/*G*, *B* represents the bulk modulus, and *G* represents the shear modulus. The calculated results are also summarized in Table 3, which indicates that AlCu_2_Mn shows high intrinsic hardness; meanwhile, AlCu_2_Hf and AlCu_2_Pm have the lowest values according to Tian’s model.

### 3.3. Electronic Properties

**Figure 2:**
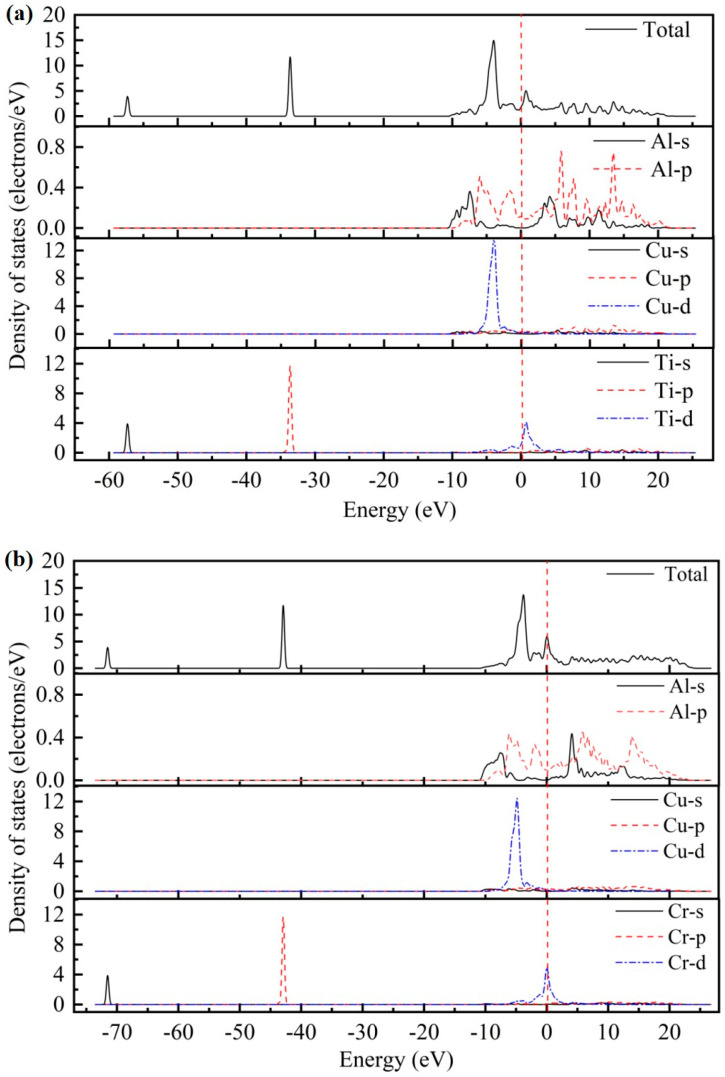

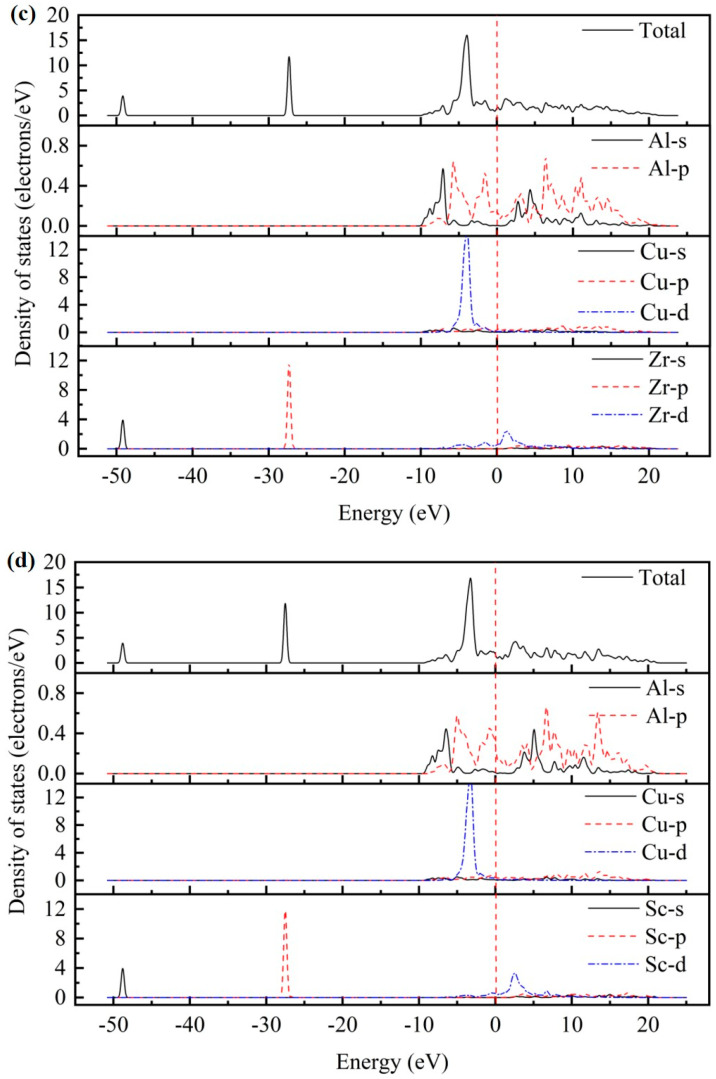

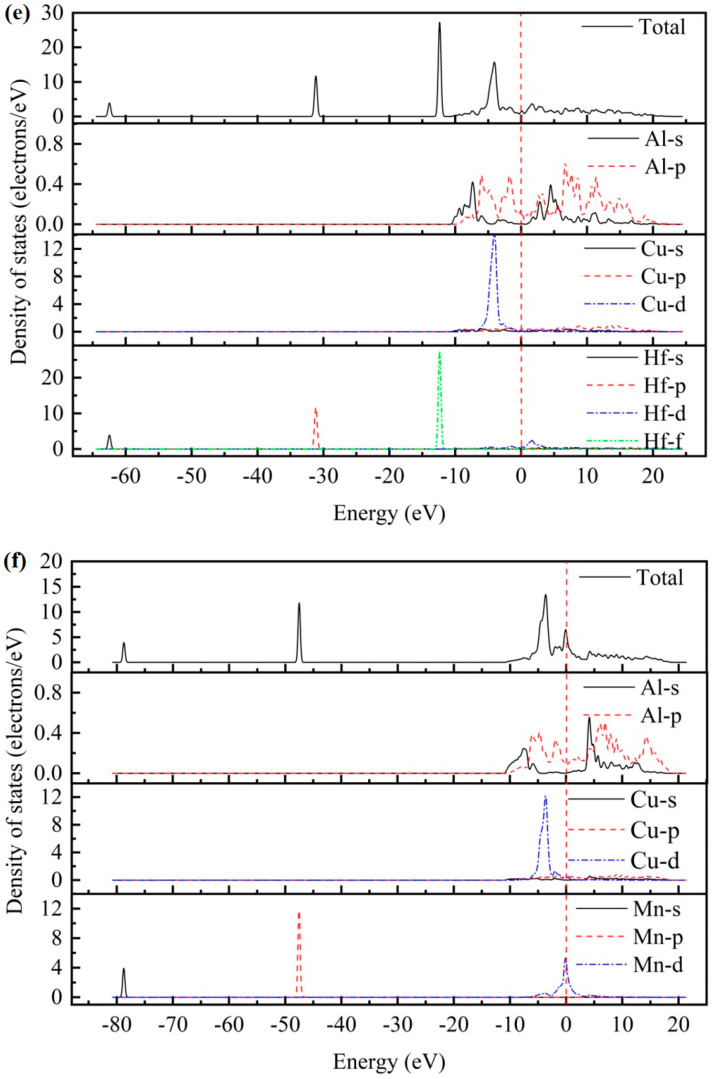

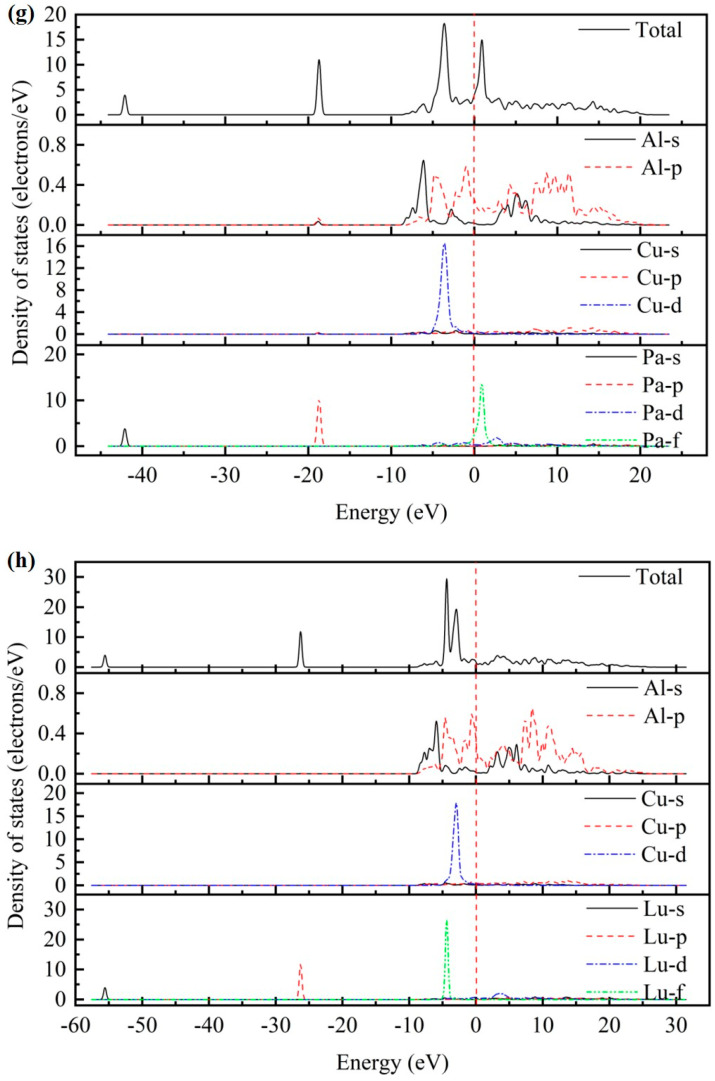

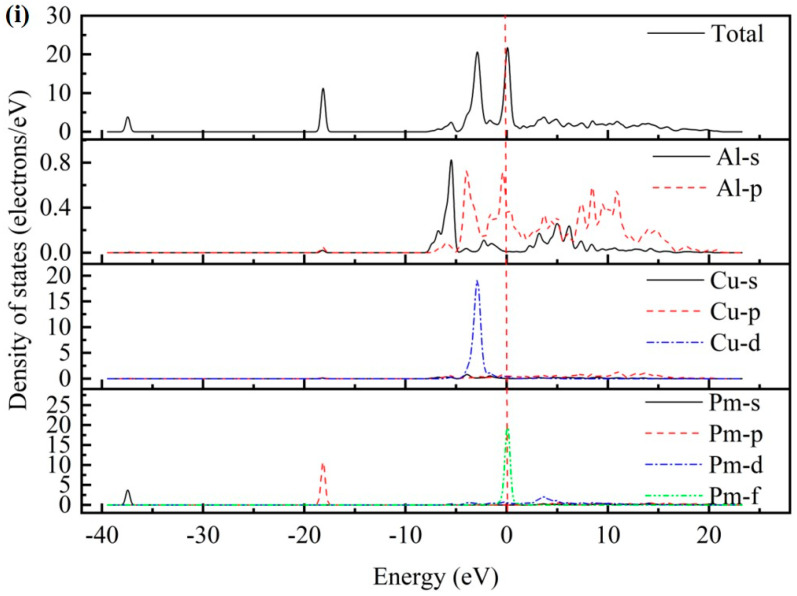
The total density of states (TDOS) and partial density of states (PDOS) of AlCu_2_M compounds. (**a**) AlCu_2_Ti, (**b**) AlCu_2_Cr, (**c**) AlCu_2_Zr, (**d**) AlCu_2_Sc, (**e**) AlCu_2_Hf, (**f**) AlCu_2_Mn, (**g**) AlCu_2_Pa, (**h**) AlCu_2_Lu and (**i**) AlCu_2_Pm.

As presented in Figure 2, the total density of states (TDOS) and partial density of states (PDOS) for the AlCu_2_M compounds have been studied. There are no band gaps at the Fermi level; it can be seen that all AlCu_2_M compounds have metallic characteristics. The Fermi energy (*E_F_*) results of AlCu_2_M (M = Ti, Cr, Zr, Sc, Hf, Mn, Pa, Lu, and Pm) compounds are 1.853 eV, 5.966 eV, 1.510 eV, 1.647 eV, 1.535 eV, 6.094 eV, 4.060 eV, 1.691 eV, and 21.083 eV, respectively. The crystal structures of AlCu_2_M are very similar, but their TDOSs differ greatly from one another. This variation is mostly due to the electronic configuration of the M (M = Ti, Cr, Zr, Sc, Hf, Mn, Pa, Lu, and Pm) elements. The highest peak of TDOS for all AlCu_2_M is roughly at −4.5 eV, except for AlCu_2_Pm. AlCu_2_Hf has the broadest pseudo-gap or quasi-gap close to the Fermi energy, indicating that it has the strongest covalent bond, which is consistent with the bulk modulus analysis.

Meanwhile, the partial densities of states (PDOS) of AlCu_2_M compounds are calculated to study the electronic structure, as shown in Figure 2. In the case of AlCu_2_M (M = Ti, Cr, Zr, Sc, Mn, Pa, and Pm), in the energy region below −10 eV, the bands are primarily provided by M-s and M-p states. For AlCu_2_M (M = Hf and Lu) compounds, the M-s, M-p, and M-f states are primarily responsible for the TDOS below −10eV. Strong hybridization is observed in the energy range of −10ev to 0 eV, and the Al-Cu covalent bond is attributed to hybridization between Al-p and Cu-d states The Al-M covalent bond is attributed to hybridization between Al-s and M-f states, which produced two sharp peaks in TDOS. This is consistent with the results of Dong et al. [10] and Pang et al. [11]. Atomic orbitals of Al-s, Al-p, and M-d states hybridize near the Fermi energy for AlCu_2_M (M = Ti, Cr, Zr, Sc, and Mn) compounds, and the Al-s and Al-p states hybridize with M-f states for the AlCu_2_M (M = Hf and Lu) compounds. It is shown that atoms of Al and M form Al-M covalent bonds and produce a second peak in the TDOS. Above Fermi energy, the Al-p states hybridize with M-d states for AlCu_2_M compounds, with the exception of the Al-p states, which hybridize with M-f states for AlCu_2_Pa. Moreover, the Al-M covalent bonds in AlCu_2_M (M = Pa, Lu, and Pm) are connected to the Al-s, Al-p, and M-f hybridization states and are stronger than those in other AlCu_2_M (M = Ti, Cr, Zr, Sc, Hf, and Mn) compounds.

### 3.4. Thermodynamic Properties

To investigate the influence of M (M = Ti, Cr, Zr, Sc, Hf, Mn, Pa, Lu, and Pm) element doping of Al-Cu alloys on their heat-conducting properties, it is necessary to study the thermodynamic properties of AlCu_2_M compounds. In this section, the Debye temperature (Θ*_D_*) and the minimum thermal conductivity (*κ_min_*) are calculated and discussed at length. Debye temperature can establish the relationship between the elastic modulus and thermophysical properties of AlCu_2_M compounds, and that is related to characterizing the strength of chemical bonds, which can be expressed as follows [41]:

(6) $$\Theta_{D}=V_{m}\frac{h}{k_{B}}{\left[\frac{3n}{4\pi }(\frac{N_{A}\rho }{M})\right]}^{\frac{1}{3}}$$

where *h* refers to Planck’s constant, *k_B_* is Boltzmann’s constant, *N_A_* is Avogadro’s number, *ρ* is the density of AlCu_2_M compounds, *M* is the molecular weight, *n* is the number of atoms per formula unit, and *V_m_* refers to the average wave velocity, which can be calculated via the following equation [42].

(7) $$V_{m}={\left[\frac{1}{3}(\frac{1}{V_{l}^{3}}+\frac{2}{V_{t}^{3}})\right]}^{-\frac{1}{3}}$$

where *V_t_* represents the traverse elastic wave velocity, and *V_l_* is the longitudinal elastic wave velocity. The values of *V_t_* and *V_l_* can be obtained by Navier’s equation [42]:

(8) $$V_{l}={\left[(B+\frac{4}{3}G)/\rho \right]}^{\frac{1}{2}}$$

(9) $$V_{t}={\left[G/\rho \right]}^{\frac{1}{2}}$$

The results of shear modulus *G* and the bulk modulus *B* are shown in Table 3. The calculated values of *ρ*, *V_t_*, *V_l_*, *V_m_*, and Θ*_D_* for the AlCu_2_M compounds are listed in Table 4. AlCu_2_Mn exhibits the highest Debye temperatures, and AlCu_2_Pm is the lowest at zero pressure and zero Kelvin. It is shown that AlCu_2_Mn has a stronger strength of chemical bonds than that of other AlCu_2_M compounds, which is consistent with the behavior of the elastic modulus mentioned above.

In general, the thermal conductivity reduces as the temperature increases, and the minimum thermal conductivity means that it reaches a minimum state in which the lattice vibration is weakest [43]. The minimum thermal conductivity (*κ_min_*) is another important thermal property that reflects the ability of materials to transfer heat. Study of the thermal conductivity of the doping elements *M* is required to determine whether or not they are advantageous for conducting heat. The *κ_min_* for Clarke’s model at high temperatures (higher than the Debye temperature) is found through the following expressions [43]:

(10) $$\kappa_{\min}^{clarke}=0.87k_{B}M_{a}^{-\frac{2}{3}}E^{\frac{1}{2}}\rho^{\frac{1}{6}}$$

(11) $$M_{a}=M/(n\cdot N_{A})$$

where the symbols of *k_B_*, *M*, *n*, *N_A_*, and *ρ* have the same meaning as used in Equation (6), and *E* is the Young’s modulus, calculated above in Section 3.2. Based on the Cahill–Pohl model [44], the equation of *κ_min_* is as follows:

(12) $$\kappa_{\min}^{cahill-pohl}=\frac{k_{B}}{2.48}{n^{\prime }}^{\frac{2}{3}}\left(V_{l}+2V_{t}\right)$$

where *k_B_* is the Boltzmann constant, *n*’ is the density of atoms per volume, and *V_t_* and *V_l_* are as above in Equations (8) and (9). The calculated results of the minimum thermal conductivity of AlCu_2_M (M = Ti, Cr, Zr, Sc, Hf, Mn, Pa, Lu, and Pm) are also listed in Table 4. The results from Clarke’s model are marginally higher than those of the Cahill–Pohl model. Table 4 shows that AlCu_2_Mn has a higher Debye temperature than the other phases and thus has the greatest thermal conductivity for both Clarke’s model and the Cahill–Pohl model. Furthermore, it can also be observed that AlCu_2_Pm has a lower *κ_min_* value than that of the others, indicating a poor heat transfer capability. The results of the above Debye temperature agree well with the calculations from the two models of *κ_min_* in this paper.

## 4. Conclusions

In summary, the structural, elastic, electronic, and thermodynamic properties of AlCu_2_M (M = Ti, Cr, Zr, Sc, Hf, Mn, Pa, Lu, and Pm) compounds have been systematically investigated using first-principles calculations based on density functional theory. Due to the achievement of the negative formation energy and the fulfillment of mechanical stability requirements, it is demonstrated that all AlCu_2_M compounds have both structural and mechanical stability. The calculated Young’s modulus *E* and hardness of AlCu_2_Mn are higher than those of the others. The Poisson’s ratio and *B*/*G* results show that all AlCu_2_M compounds are ductile. The DOS of AlCu_2_M compounds is mainly contributed by Al-s states, Al-p states, Cu-d states, M-d states (M = Ti, Cr, Zr, Sc, and Mn), or M-f states (M = Hf, Pa, Lu, and Pm), and strong bonding forms between Al atoms and Cu atoms. Meanwhile, the Debye temperature is determined by the average wave velocity; AlCu_2_Mn and AlCu_2_Sc have higher Debye temperatures than the others. The minimum thermal conductivity is also calculated from the average elastic wave velocity; AlCu_2_Mn and AlCu_2_Sc have larger minimum thermal conductivities, which are consistent with the Young’s modulus values. This work may be expanded to other intermetallic compounds and offers some alloying recommendations for enhancing the ductility, hardness, and thermal conductivity of AlCu_2_M compounds.

## Figures and Tables

**Table 1:** Calculated and experimental lattice parameters α (Å), equilibrium volume V_0_ (Å^3^), and formation energy Δ*H* (eV/atom) for AlCu_2_M compounds.

Compounds	Method	Lattice Constants	*V*_0_ (Å^3^)	Δ*H* (eV/Atom)	Ref.
		*α* (Å)			
AlCu_2_Ti	Cal.	6.037	220.043	−4.985	Present
	Cal.	6.032	219.467	−4.235	[10]
	Exp.	6.024	218.570	-	[20]
AlCu_2_Cr	Cal.	5.869	202.160	−5.283	Present
	Cal.	5.891	204.429	-	[11]
	Exp.	5.809	196.020	-	[21]
AlCu_2_Zr	Cal.	6.256	244.879	−5.167	Present
	Cal.	6.256	244.827	−4.551	[10]
	Exp.	6.216	240.210	-	[20]
AlCu_2_Sc	Cal.	6.223	241.031	−4.546	Present
	Cal.	6.243	243.327	-	[11]
	Exp.	6.199	238.210	-	[22]
AlCu_2_Hf	Cal.	6.220	240.654	−5.070	Present
	Cal.	6.210	239.535	−4.451	[10]
	Exp.	6.172	235.110	-	[23]
AlCu_2_Mn	Cal.	5.850	200.283	−5.139	Present
	Cal.	5.817	196.844	−3.419	[10]
	Exp.	5.968	212.560	-	[24]
AlCu_2_Pa	Cal.	6.465	270.253	−5.534	Present
	Cal.	6.47	270.63	-	[25]
AlCu_2_Lu	Cal.	6.373	258.859	−4.720	Present
	Cal.	6.29	249.33	-	[25]
AlCu_2_Pm	Cal.	6.539	279.688	−4.195	Present
	Cal.	6.53	278.64	-	[25]

**Table 2:** Calculated elastic constants (*C_ij_*, in GPa) for the AlCu_2_M compounds.

Compounds	Elastic Constants *C_ij_*			Ref.
	*C*_11_	*C*_12_	*C*_44_	
AlCu_2_Ti	172.476	149.587	122.355	Present
	163.314	120.827	96.918	[10]
	144.486	124.338	97.943	[11]
AlCu_2_Cr	133.213	124.467	100.126	Present
	157.5	115.3	62.7	[30]
AlCu_2_Zr	167.383	133.152	70.846	Present
	157.504	115.305	62.685	[10]
AlCu_2_Sc	135.729	77.034	68.615	Present
	155.252	78.727	75.987	[10]
AlCu_2_Hf	166.777	152.559	72.617	Present
	171.570	116.888	68.685	[10]
AlCu_2_Mn	198.051	114.279	124.332	Present
	225.069	121.565	107.411	[10]
AlCu_2_Pa	157.043	75.478	22.908	Present
AlCu_2_Lu	126.094	64.315	42.077	Present
AlCu_2_Pm	117.970	57.364	3.993	Present

**Table 3:** The calculated bulk, shear, and Young’s modulus (GPa), Poisson ratio *ν*, *B*/*G*, and hardness *H_VT_* (GPa) for AlCu_2_M compounds at zero pressure.

Compounds	Elastic Modulus (GPa)			*ν*	*B*/*G*	*H_VT_* (GPa)	Ref.
	*B*	*G*	*E*				
AlCu_2_Ti	157.217	51.540	126.888	0.352	3.050	4.220	Present
	134.989	54.481	-	-	2.478	-	[10]
	131.054	42.307	120.247	0.354	3.098	-	[11]
	129.22	37.38	102.278	0.368	3.457	-	[35]
AlCu_2_Cr	127.383	36.042	90.967	0.397	4.501	2.771	Present
	144.898	29.436	82.707	0.405	4.922	-	[11]
AlCu_2_Zr	157.047	38.477	99.224	0.387	4.082	2.464	Present
	129.371	41.237	-	-	3.137	-	[10]
AlCu_2_Sc	142.302	57.078	135.106	0.324	2.493	5.705	Present
	104.235	57.696	146.127	0.266	1.807	-	[11]
AlCu_2_Hf	176.664	36.197	95.541	0.405	4.881	1.925	Present
	135.115	47.971	-	-	2.817	-	[10]
AlCu_2_Mn	142.203	80.458	175.265	0.262	1.767	10.757	Present
	156.066	80.521	-	-	1.938	-	[10]
AlCu_2_Pa	102.667	28.918	73.039	0.372	3.550	2.359	Present
AlCu_2_Lu	84.908	37.177	86.331	0.309	2.284	4.653	Present
AlCu_2_Pm	77.566	10.318	28.433	0.436	7.518	0.485	Present

**Table 4:** The density *ρ* (g/cm^3^), elastic wave velocity (m/s), Debye temperature Θ*_D_* (K), and the minimum thermal conductivity *κ*_min_ (W/m·K) of AlCu_2_M compounds.

Compounds	*ρ*	*V*			Θ*_D_*	*κ_min_*	
		*V_t_*	*V_l_*	*V_m_*		Clark	Cahill-Pohl
AlCu_2_Ti	6.619	2790.550	5842.669	3768.708	481.329	0.968	0.227
AlCu_2_Cr	6.678	2323.171	5125.528	3165.179	402.728	0.810	0.189
AlCu_2_Zr	7.175	2315.789	5388.834	3179.895	391.013	0.762	0.175
AlCu_2_Sc	6.012	3081.312	6027.450	4106.714	510.415	0.992	0.238
AlCu_2_Hf	9.866	1915.424	4774.732	2652.930	327.756	0.644	0.146
AlCu_2_Mn	6.932	3406.952	5999.286	4428.178	567.786	1.120	0.279
AlCu_2_Pa	9.464	1748.006	3862.900	2382.164	276.413	0.507	0.117
AlCu_2_Lu	8.443	2098.392	3990.930	2779.609	327.185	0.600	0.145
AlCu_2_Pm	7.150	1201.278	3573.853	1690.776	193.957	0.356	0.079

## Data Availability

The original contributions presented in the study are included in the article, further inquiries can be directed to the corresponding author.
